# Protocol for a partially nested randomized controlled trial to evaluate the effectiveness of the Scleroderma Patient-centered Intervention Network Support Group Leader EDucation (SPIN-SSLED) Program

**DOI:** 10.1186/s13063-019-3747-z

**Published:** 2019-12-12

**Authors:** Brett D. Thombs, Kylene Aguila, Laura Dyas, Marie-Eve Carrier, Claire Fedoruk, Linda Horwood, Mara Cañedo-Ayala, Maureen Sauvé, Linda Kwakkenbos, Vanessa L. Malcarne, Ghassan El-Baalbaki, Sandra Peláez, Kerri Connolly, Marie Hudson, Robert W. Platt, SPIN-SSLED Support Group Advisory Team, Stephen Elrod, Catherine Fortuné, Karen Gottesman, Karen Nielsen, Ken Rozee, Amy Gietzen, Michelle Richard, Nancy Stephens

**Affiliations:** 1 Lady Davis Institute for Medical Research, Jewish General Hospital, Montreal, QC Canada; 2 Department of Psychiatry, McGill University, Montreal, QC Canada; 3 Department of Epidemiology, Biostatistics, and Occupational Health, McGill University, Montreal, QC Canada; 4 Department of Medicine, McGill University, Montreal, QC Canada; 5 Department of Psychology, McGill University, Montreal, QC Canada; 6 Department of Educational and Counselling Psychology, McGill University, Montreal, QC Canada; 7 Biomedical Ethics Unit, McGill University, 4333 Cote Ste Catherine Road, Montreal, QC H3T 1E4 Canada; 8 Scleroderma Foundation Michigan Chapter, Southfield, MI USA; 9 Scleroderma Society of Ontario and Scleroderma Canada, Hamilton, ON Canada; 10 Department of Clinical Psychology, Behavioural Science Institute, Radboud University, Nijmegen, the Netherlands; 11 Department of Psychology, San Diego State University, California, USA; 12 San Diego Joint Doctoral Program in Clinical Psychology, San Diego State University/University of California, California, USA; 13 Department of Psychology, Université du Québec à Montréal, Montreal, QC Canada; 14 Scleroderma Foundation, Danvers, MA USA

**Keywords:** Patient education, Peer support, Feasibility trial, Scleroderma, Support groups, Systemic sclerosis

## Abstract

**Background:**

Some people with rare diseases rely on peer-led support groups for disease-specific education and emotional and practical support. Systemic sclerosis (SSc), or scleroderma, is a rare autoimmune connective tissue disease. Many people with SSc cannot access support groups, and, when support groups exist, they may not be sustained due to challenges that could be addressed via leader training. The Scleroderma Patient-centered Intervention Network (SPIN), along with SSc patient organization partners, developed a training program for SSc patient support group leaders, the Scleroderma Support group Leader EDucation (SPIN-SSLED) Program. We recently completed a feasibility trial in which we successfully delivered the program to two groups of support group leaders who reported a high level of satisfaction with the program and its delivery. The primary objective of the full-scale SPIN-SSLED trial is to evaluate the effect of the program on support group leaders’ self-efficacy for carrying out their leadership role. Secondary objectives include evaluating effects on leader burnout, leader satisfaction (participation efficacy), and emotional distress.

**Methods/design:**

The SPIN-SSLED trial is a pragmatic randomized controlled trial (RCT) in which 180 support group leaders will be randomly allocated to training groups of 6 participants each or to a waitlist control. We will use a partially nested RCT design to reflect dependence between individuals in training groups, but not in the waitlist control. Participants allocated to the training program will receive the 13-module SPIN-SSLED Program, delivered via webinar over the course of 3 months in weekly 60–90-min sessions. The primary outcome is leader self-efficacy, measured by the Scleroderma Support Group Leader Self-efficacy Scale post-intervention. Secondary outcomes are leader self-efficacy at 3 months post-intervention, and leader burnout, volunteer job satisfaction (participation efficacy), and emotional distress post-intervention and at 3 months post-intervention.

**Discussion:**

The SPIN-SSLED trial will test whether a training program for SSc patient support group leaders increases the self-efficacy of group leaders to carry out leadership tasks. The program has the potential to significantly improve the effectiveness and sustainability of existing SSc support groups, to increase the number of available support groups, and to be adapted for other chronic diseases.

**Trial registration:**

ClinicalTrials.gov, NCT03965780. Registered on 29 May 2019.

## Background

People with rare diseases face the same challenges as those with more common diseases plus other unique challenges, including limited education on disease and a lack of specialized support options [1–5]. Professionally organized support services for people with common diseases are often available through the healthcare system [6, 7], but are not typically available in rare diseases [8]. As a result, many people with rare diseases look to peer-led support groups for disease-specific education and support [9–14]. Support groups provide important benefits to people with burdensome medical conditions, based on the principle that people who face similar challenges can empower one another through emotional and practical support [7, 14]. Support groups may be held face to face or online, led by professionals or peers, and have a structured or unstructured format. Activities typically involve an educational or information-sharing component and the exchange of emotional and practical support [7, 11].

Peer support interventions, including support groups, have been found to increase positive health behaviors, self-efficacy for disease management, and mental health [15–17]. Disease-specific peer support services, however, are often not accessible to people with rare diseases [8]. One reason is that there are major obstacles to evaluating and delivering organized support (e.g., support groups, peer-to-peer support) for people with rare diseases. We previously searched PubMed using the names of the approximately 7000 rare diseases listed in Orphanet’s Orphadata database [18] but did not find any trials of organized support programs for patients with any rare disease [19].

Peer-led support groups play an important role for many people with systemic sclerosis (SSc), or scleroderma. SSc is a rare, chronic, autoimmune disease characterized by vasculopathy and excessive collagen production [20, 21]. Onset typically occurs between the ages of 30 and 50 years, and approximately 80% of people with SSc are women [20, 21]. SSc can affect multiple organ systems, including the skin, lungs, gastrointestinal tract, and heart. Common manifestations include Raynaud’s phenomenon, skin thickening, dyspnea and cough, gastroesophageal reflux and other gastrointestinal symptoms [20, 21]. People with SSc commonly experience hand function and mobility limitations, pain, fatigue, sleep problems, pruritus, depression, and body image distress from disfigurement (e.g., skin tightening, pigment changes, hand contractures, telangiectasias) [22–28]. Disease presentation is extremely heterogeneous, and the course of the disease is highly unpredictable [20, 21].

Currently, there are over 250 leaders and co-leaders affiliated with Scleroderma Canada and Canadian provincial organizations, the Scleroderma Foundation of the United States, Scleroderma & Raynaud’s UK, Scleroderma Australia and Australian state organization, and Scleroderma New Zealand; almost all of these leaders are people with SSc [29–32]. Many people with SSc, however, cannot access support groups, and many initiated support groups are not sustained due to challenges that could be addressed via leader training [10–13, 29–32]. Currently, there are only a handful of support groups offering support delivered via teleconference or videoconference [29–32]. As such, most people with SSc must live close enough to a local group and be able to travel to participate [11, 12]. However, when local groups do exist, they are sometimes not sustained due to the leader’s health or to issues related to untrained peer leaders. Some patients have reported that they prefer not to attend SSc support groups because the group in their area is poorly organized or is overly negative [11, 12].

Research in SSc and other diseases, including cancer, has established that leading a support group poses significant challenges and a high level of burden for patient leaders, often resulting in burnout [13, 33, 34]. Peer leaders of illness-based support groups report challenges that include practical difficulties, such as a lack of resources or poor coordination with medical professionals; difficulties with group leadership tasks, such as managing complex group dynamics or dealing with the worsening health or death of group members; and personal challenges, such as balancing personal and group demands, preventing burnout and stress, and managing one’s own health condition while supporting others [11–13, 33–35]. These challenges are magnified for peer leaders of rare-disease support groups, who also face logistical problems related to small numbers of potential group members, even in urban settings, and limited support from healthcare and patient organizations, which are not as well-resourced as organizations for people with more common diseases, such as cancer, heart disease, or arthritis [13, 19].

The Scleroderma Patient-centered Intervention Network (SPIN) partnered with SSc patient organization leaders and with a Support Group Leader Advisory Team that was formed by SPIN to develop the Scleroderma Support group Leader EDucation (SPIN-SSLED) Program. The program is a 3-month group videoconference training program, designed to improve skills and self-efficacy, reduce burden, and reduce emotional distress among support group leaders. By providing key knowledge and skills, the SPIN-SSLED Program may improve the ability of SSc peer support group leaders to lead sustainable, effective support groups; reduce burden on leaders; and encourage new leaders to set up support groups where none exist, locally or via the Internet.

The program is designed to be delivered by videoconference because in rare diseases, including SSc, support group leaders are widely dispersed geographically. Videoconferencing has been successfully used to train educators, therapists, and other health service providers [36–40]. Systematic reviews have found that training healthcare service providers via videoconferencing achieves similar learning outcomes to traditional face-to-face models [39, 40].

We previously conducted a systematic review of trials that have evaluated the effects of training programs for patient leaders of illness-based support groups on the competency, self-efficacy, burden, and emotional well-being of group leaders [41]. Only one randomized controlled trial (RCT) met inclusion criteria [42]. That study evaluated confidence and self-efficacy of cancer support group leaders randomized to either 4-month-long high-resource (*N* = 29; website, discussion forum, 2-day face-to-face training) or low-resource (*N* = 23; website, discussion forum) interventions. The RCT did not find evidence that the high-resource program was more effective. However, the trial was substantially underpowered, not enough information was provided to determine intervention content or how it was delivered, and risk of bias was high due to methodological limitations. A recent update of the systematic review did not identify any additional trials [43].

We recently conducted a feasibility trial of the SPIN-SSLED Program (ClinicalTrials.gov, NCT03508661) that involved delivery of the SPIN-SSLED Program to two training groups of five participants each [44]. Scleroderma Canada and the Scleroderma Foundation each provided the names of 6 potential participants who were current support group leaders; all 12 agreed to participate in the program. We enrolled 10 participants initially and wait-listed the other 2, but 1 participant was hospitalized before the trial began; therefore, we added 1 participant who had been waitlisted. Participant attendance was high for the 13 sessions (95%; 123 of 130 possible sessions). All 10 participants completed all baseline and post-intervention measures, including an interview that addressed topics related to usability, understandability, organization and clarity of the SPIN-SSLED program. No sessions were missed or delayed due to technological difficulties, and time spent on technological support for participants from our team was < 2 h for the entire program [44].

In the feasibility trial, the pre-intervention mean (standard deviation (SD)) total score on a measure of support group leader self-efficacy, the Scleroderma Support Group Leader Self-efficacy Scale (SSGLSS) [45], the primary outcome for the planned full-scale trial, was 124.4 (22.0). Post-training, the mean (SD) total score increased to 159.2 (17.1), indicating increased participant self-efficacy. The standardized mean difference effect size was 1.7. Items are scored on a scale of 1–6; the average item score increase pre-post training was 1.1 points [44]. Participant satisfaction was high. The mean (SD) post-training score on the Client Satisfaction Questionnaire-8 (CSQ-8) [46] was 30.6 (2.2). On a per item basis, the mean item score was 3.8 (item range 1–4). In post-intervention interviews, there were relatively minor suggestions for improving the program, and feedback was extremely positive. The overall mean rating given by participants for the program was 9.4/10, and all 10 participants indicated they would recommend the program to other support group leaders.

The planned full-scale SPIN-SSLED trial will be a parallel-group, partially nested RCT (PN-RCT) with a 1:1 allocation ratio that will test the superiority of the SPIN-SSLED Program to a waitlist control group for improving support group leaders’ self-efficacy (defined as their perceived ability to carry out actions needed to be successful in support group leadership) [47] and improving leader satisfaction and reducing leader burnout (including emotional exhaustion and disengagement) [48] and emotional distress. The reason that we will use a waitlist control group that will receive the program post-trial is that our patient organization partners are invested in providing the training program, regardless of trial outcomes, for reasons of organizational liability and in order to support their support group leader community, the members of which have expressed a strong desire to receive training.

The primary objective of the SPIN-SSLED trial is to evaluate the effect of the SPIN-SSLED Program on support group leaders’ self-efficacy, measured by the SSGLSS [45] post-intervention. Secondary objectives are to evaluate the program’s effects on (1) the SSGLSS [45] at 3 months post-intervention; (2) burnout, measured by the Oldenburg Burnout Inventory (OLBI) [48, 49] post-intervention and 3 months post-intervention; (3) leader satisfaction that leading a support group is helping others, measured by the Participation Efficacy subscale of the Volunteer Satisfaction Index (VSI) [50] post-intervention and 3 months post-intervention; and (4) emotional distress, measured by the Patient Health Questionnaire-8 (PHQ-8) [51, 52] post-intervention and 3 months post-intervention. In addition, we will evaluate participant satisfaction with the program among those randomized to the program via the CSQ-8 post-intervention [46].

## Methods

The planned trial has been registered (ClinicalTrials.gov, NCT03965780), and the present protocol follows recommendations for reporting from the Standard Protocol Items: Recommendations for Interventional Trials (SPIRIT) 2013 statement [53]. Results of the trial will be reported in accordance with standards articulated in the Consolidated Standard of Reporting Trials (CONSORT) statement [54] and CONSORT extensions for nonpharmacologic trials [55], cluster trials [56], pragmatic trials [57], and e-health trials [58]. Initial participant enrollment is planned for July and August 2019 with randomization of participants into the first wave of training and waitlist control groups scheduled for September 2019.

The trial will be a pragmatic RCT that tests whether the SPIN-SSLED Program improves support group leader outcomes compared to leaders assigned to a waitlist control. Pragmatic RCTs are intended to replicate real-world conditions and support a decision on whether an intervention should be provided [57, 59, 60].

Support group leaders randomly assigned to the SPIN-SSLED Program will be clustered into training groups. Members of each training group will interact during videoconference training modules. Support group leaders randomly assigned to the waitlist control will not be clustered; they will only complete trial measures. A standard cluster RCT design is used when interventions are delivered to groups, rather than individuals, in order to account for dependence between individuals within clusters [56]. The SPIN-SSLED trial will need to account for clustering in the intervention arm but not the control arm. Thus, we will use a PN-RCT trial design [61]. The PN-RCT design is a hybrid between a conventional RCT with individual participant randomization and a cluster RCT, in which pre-existing clusters (e.g., primary care practices) are randomized to intervention or control arms. In the PN-RCT design, analyses account for dependence within intervention arm clusters but treat participants assigned to the control arm individually as in a conventional RCT [61, 62]. Although less common in medical research, PN-RCTs are used extensively in educational and behavioral research [61].

### Study setting and eligibility

Trial participants will include current support group leaders and candidate leaders who are affiliated with Scleroderma Canada and Canadian provincial organizations, including Sclérodermie Québec; the Scleroderma Foundation in the USA; Scleroderma & Raynaud’s UK; Scleroderma Australia and Australian state organizations; and Scleroderma New Zealand. To be eligible, support group leaders must be identified by one of our partner organizations as a current or candidate leader, must be able to use the Internet to access training sessions, must indicate that they would be comfortable with participating in sessions offered in English or French, and must be able to complete study questionnaires online in English or French. In addition to these requirements, we will only enroll one support group leader per support group in order to avoid contamination, whereby leaders in the waitlist arm could receive training materials from their support group co-leader. In the case where there are multiple leaders for a single existing support group, the co-leaders must come to a decision together on who they would like to be the primary leader and prioritized for enrollment. The leader(s) designated as secondary will only be enrolled if the primary leader in the group must drop out prior to random selection and allocation or does not have any day and time availabilities that match those offered as part of the trial and, thus, will not be eligible for random selection and allocation. Leaders who are designated as secondary and who do not undergo training will be placed in the waitlist to undergo training post-trial. All participants were free to access any care resources or other interventions made available to them throughout the course of the trial.

### The SPIN-SSLED Program

The SPIN-SSLED Program uses problem-based learning, which is a learner-centered approach that integrates theory and practice by providing necessary knowledge and skills, presenting complex, real-world problems, and working to identify approaches to solving problems [63, 64]. Each module, or learning session, will introduce a topic and provide information on the topic. In modules that involve managing group or individual interactions, videos recorded with members of the SPIN Support Group Advisory Team will show SSc support group leaders faced with a problem or situation similar to those that training group participants may encounter in their role as a support group leader. Then, there will be a guided discussion among training group participants about possible approaches and solutions.

The SPIN-SSLED Program will be offered in English or in French; the English version of the Program was translated into French by a research assistant and then reviewed for consistency by bilingual research team members. The program includes 13 modules that are delivered via videoconference over the course of the 3-month program in weekly 60–90-min sessions. Module topics include (1) The leader’s role; (2) Starting a support group; (3) Structuring a support group meeting; (4) Scleroderma 101; (5) Successful support group culture; (6) Managing support group dynamics; (7) Loss and grief: The support group leader; (8) Loss and grief: supporting group members; (9) Advertising and recruitment for the support group; (10) The continuity of the group; (11) Supporting yourself as a leader; (12) Remote support groups; and (13) Transitions in support groups. See Additional file 1 for an overview of module content.

All English-language SPIN-SSLED training groups will be facilitated by a single instructor who assisted in the development of the SPIN-SSLED training program and delivered the program during the feasibiilty trial. She is a trained social worker with 28 years of total experience and over 10 years of experience working in SSc. The French-language training groups will be facilitated by a single instructor who is a recent graduate of the SPIN-SSLED program and has led a support group in Quebec for the last 5 years. Instructors will guide each session using the SPIN-SSLED instructor manual, which is based on the program manual but includes guidance on introducing material and discussion prompts. Participants will receive a SPIN-SSLED program manual that summarizes didactic material from sessions. Based on our previous experience and consistent with previous trials of videoconference training, six support group leaders will be assigned to each training group to maximize effective interaction and participation [36, 37]. Training sessions will be delivered using the GoToMeeting® videoconferencing platform, a high-performance platform that has been used successfully in similar applications [38, 65] and that was used successfully in the SPIN-SSLED Feasibility trial [44]. In addition to the videoconference sessions, participants will have access to a secure, monitored SPIN-SSLED online forum to interact with other participants about program content and a resource center with video presentations for patients made by SSc expert physicians and other material that they can use for their support groups.

All SPIN-SSLED sessions will be video-recorded and audited for fidelity to the program manual by two members of the research team. We will use standard methods for evaluating intervention fidelity [66], including observation of entire sessions for a randomly selected sample of 25% of sessions. Raters will evaluate adherence to each session’s goals and content. Consistent with best-practice recommendations for assessing treatment fidelity [66], this will be done using a checklist based on a standardized format adapted for the specific components of the SPIN-SSLED Program manual.

Participants may choose to discontinue their participation in the training sessions at any time. We do not envision, however, the need to modify the intervention or intervention assignment for any participants or to discontinue their participation in the program.

### Outcomes

The primary outcome analysis will compare SSGLSS [45] scores between group leaders allocated to the SPIN-SSLED Program versus the waitlist control post-intervention. The SSGLSS is a 32-item scale designed to assess SSc support group leader confidence to successfully perform leader tasks (e.g., organizational skills), manage group and interpersonal interactions, and balance group leadership and self-care needs. The measure reflects the core educational content of the SPIN-SSLED Program. It utilizes a 6-point Likert scale ranging from 1 (strongly disagree) to 6 (strongly agree), with higher total scores indicating greater self-efficacy. Prior to the development of the SSGLSS, there were no measures of support group leader self-efficacy. We developed the SSGLSS with our Support Group Leader Advisory Team, translated it into French using an accepted forward-backward translation method, [67] and validated it in two samples of SSc support group leaders (*N* = 102, *N* = 55). We found that it had good internal consistency (Cronbach’s alpha 0.96 and 0.95) and hypothesis-consistent convergent validity with a burnout measure, the OLBI [45]. In our feasibility trial [44], SSGLSS pre-post difference was large among participants (standardized mean difference = 1.7; 1.1 point difference per item), suggesting sensitivity to change.

Secondary outcomes include the SSGLSS 3 months post-intervention and other outcome measures post-intervention and 3 months post-intervention. Leader burnout will be measured by the OLBI, which assesses exhaustion and disengagement due to burnout and has been validated in diverse populations (16 items, 4-point scale from 1 = strongly disagree to 4 = strongly agree) [48, 49]. The OLBI was initially designed for work-related burnout but has been adapted for numerous settings and in multiple countries and languages [68]. Our research team revised the wording of each of the OLBI items in the English and French [69] versions to reflect the support group environment rather than a work environment (e.g., “I find my work to be a positive challenge” was revised to “I find my role as a support group leader to be a positive challenge”). The OLBI has a two-factor structure (exhaustion and disengagement) with good measurement properties [48, 49, 68]. Higher scores on each factor indicate higher levels of exhaustion and disengagement. Internal consistency reliability (Cronbach’s alpha) in patients with SSc was 0.84 for exhaustion and 0.80 for disengagement [45]. Leader satisfaction (participation efficacy) will be measured using a modified version of the participation efficacy subscale of the VSI. The original version of the VSI was validated using a sample of volunteers (*N* = 327) and was found to be reliable and constructually valid [50]. As in other studies [70, 71], we modified the wording of some of the items to reflect participants’ volunteer role as support group leaders. The participation efficacy subscale asks respondents to indicate their level of satisfaction on 7 items using a 7-point Likert scale from 1 (very dissatisfied) to 7 (very satisfied). Emotional distress will be assessed using the PHQ-8 [51, 52]. PHQ-8 items measure depressive symptoms over the last 2 weeks on a 4-point scale, ranging from 0 (not at all) to 3 (nearly every day) with higher scores indicating more depressive symptoms. The PHQ-8 performs equivalently to the PHQ-9 [51], which is a valid measure of depressive symptoms in patients with SSc [52]. Leader burnout, participation efficacy, and emotional distress will be measured only among trial participants who already lead support group at the time of trial enrollment. The PHQ-8 is available in French and English. Participant satisfaction with the SPIN-SSLED Program among those allocated to the training program will be evaluated usingith the CSQ-8 [46], a standardized measure that is used to assess satisfaction with health services. Items are scored on a Likert scale from 1 (low satisfaction) to 4 (high satisfaction) with total scores ranging from 8 to 32. The CSQ-8 has been widely validated across a range of populations [46] and is available in French.

### Sample size

We identified several meta-analyses that have evaluated self-efficacy in terms of knowledge acquisition and confidence in implementing skills acquired in training programs. A 2016 Cochrane review reported a standardized mean difference effect size of 0.87 for four educational interventions designed to change knowledge of sickle cell disease among patients and caregivers (standardized mean difference = 1.12 with an outlier study removed) [72]. Several other meta-analyses have reported effect sizes of between 0.58 and 0.94 [73–76]. In the SPIN-SSLED feasibility trial, which only included 10 participants, the pre-post change in self-efficacy for carrying out leadership tasks was 1.7 [44]. For an assumed effect size of 0.70, a two-tailed test with α = 0.05, and an intra-class correlation coefficient (ICC) of 0.05, a sample size of 75 participants would provide ≥ 80% power for self-efficacy for carrying out leader tasks. There was no loss to follow up in our feasibility trial. Assuming 20% loss to follow up in the proposed trial, we would need to randomize 94 support group leaders. We believe that this is a conservative power and sample size estimate. First, based on previous systematic reviews and on the results of our feasibility trial, we believe that the true effect size is likely larger than 0.70. Second, in cluster RCTs, ICC values for individual patient outcomes are typically lower than our 0.05 estimate, even when different interveners are involved [77–79], and we will use the same trainer across groups in each language. If the true ICC is lower than our 0.05 estimate, this will result in greater power than estimated. Third, there was no loss to follow up in our feasibility trial, and in our previous completed studies in SSc that required follow up, loss to follow up has been 10% or less [25, 80].

For the secondary outcomes, burnout and emotional distress, based on published meta-analyses, a standardized mean difference effect size of 0.50 represents a clinically meaningful effect size for improvement that has been achieved in training programs for managers, caregivers of chronically ill patients, and parents of children with difficult behavior [73, 81–83]. This is also considered a clinically meaningful effect size for patient-reported health outcomes, including depressive symptoms [84]. For effect size of 0.50, a two-tailed test with α = 0.05, and an ICC of 0.05, a sample of 146 participants would provide ≥ 80% power for both self-efficacy and patient-reported health outcomes. Assuming 20% loss to follow up in the proposed trial, we would need to randomize 182 support group leaders. We did not estimate power for leader satisfaction.

Members of our Support Group Leader Advisory Team and our patient organization partners have emphasized the importance of evaluating the trial’s planned secondary outcomes. Thus, we will attempt to enroll 180 participants total (15 training groups of 6 participants; 90 participants in the waitlist control) in order to have sufficient power to adequately evaluate secondary outcomes.

### Recruitment

At the initiation of the trial and prior to random selection and allocation for specific training and waitlist groups, patient organization partners from Scleroderma Canada and Canadian provincial organizations, the Scleroderma Foundation in the USA, Scleroderma & Raynaud’s UK, Scleroderma Australia and Australian state organizations, and Scleroderma New Zealand will contact group leaders to describe the SPIN-SSLED Program and will provide the SPIN team with a list of eligible support group leaders. This recruitment phase is planned to begin in July 2019.

SPIN-SSLED personnel will then send an email invitation with a link to a Qualtrics survey containing the consent form, a demographic questionnaire with information about participants’ support group experience (e.g., years of experience or candidate leader), and questions on the days and times when the interested group leaders could attend training sessions. In addition to describing the study, the consent form will explain (1) that some group leaders who enroll in the study will be randomly selected every 3 months to participate in the SPIN-SSLED Program and that others will be allocated to a waitlist; (2) that participants randomized to participate in the program plus those allocated to the waitlist will complete measures online at the time of randomization, post-intervention, and 3 months post-intervention; (3) that, depending on the number of leaders who enroll, it is possible that some group leaders will not be selected to receive the training nor be asked to complete trial measures as part of the control group; and (4) that enrolled participants who do not receive the training as part of the trial, either because they are selected for the waitlist or because they are not selected for the training group or the waitlist, will be offered the training post-trial per our agreement with our partner patient organizations. At the initiation of the trial, we will send up to three emails, one per week, to leaders who do not respond to the initial email or enroll in the trial. For existing support groups where there are co-leaders, one will be specified as the primary participant and any others as secondary; only one leader per support group will be eligible for inclusion in the trial, and the secondary leaders will only be enrolled if the primary leader day and time availabilities do not match those that are able to be provided in the trial. All interested leaders will be provided contact information for SPIN-SSLED trial personnel, who will answer any questions they may have during the consent process and over the course of the trial.

We can feasibly deliver the program to 3 training groups simultaneously: thus, to deliver the 15 planned training groups, the intervention will be delivered in five “waves” with 3 training groups of 6 participants each per wave, plus 18 participants randomized to the waitlist control per wave. Prior to starting a new wave, we will email participants who have not yet been selected for participation in a prior wave to allow them to update their available days and times. We will then determine characteristics (language, day, time) of the training groups that are needed for the new wave and initiate random selection of participants and random allocation.

To ensure that we will achieve adequate enrollment to reach our target sample size, we are working closely with partner patient organizations, who will inform potential participants of the trial and will emphasize to their affiliated support group leaders the organizations are partners in the trial. They will inform affiliated leaders that participation will allow them to be certified as trained support group leaders and that the organizations plan to require the training in the future.

We will also advertise the trial through SPIN’s active Facebook and Twitter media and on the SPIN website so that potential participants and group leaders may contact their patient organizations. Additionally, a webpage [85] has been created that features a brief introductory video on the SPIN-SSLED Program, video and written testimonials from support group leaders who participated in the SPIN-SSLED Feasibility trial, and information about the program structure and content. Contact information will be provided for anyone who is interested in participating in the trial. We routinely have the opportunity to present at international, national, provincial, and local events, and we will present information on the SPIN-SSLED Program and trial at these events. We also have the opportunity to contribute to the newsletters of our patient organization partners, where we can similarly feature SPIN-SSLED. Any potential participants identified through these methods will be referred to their patient organization to ensure that they have the organization’s support to participate.

### Random selection and allocation

**Fig. 1:**
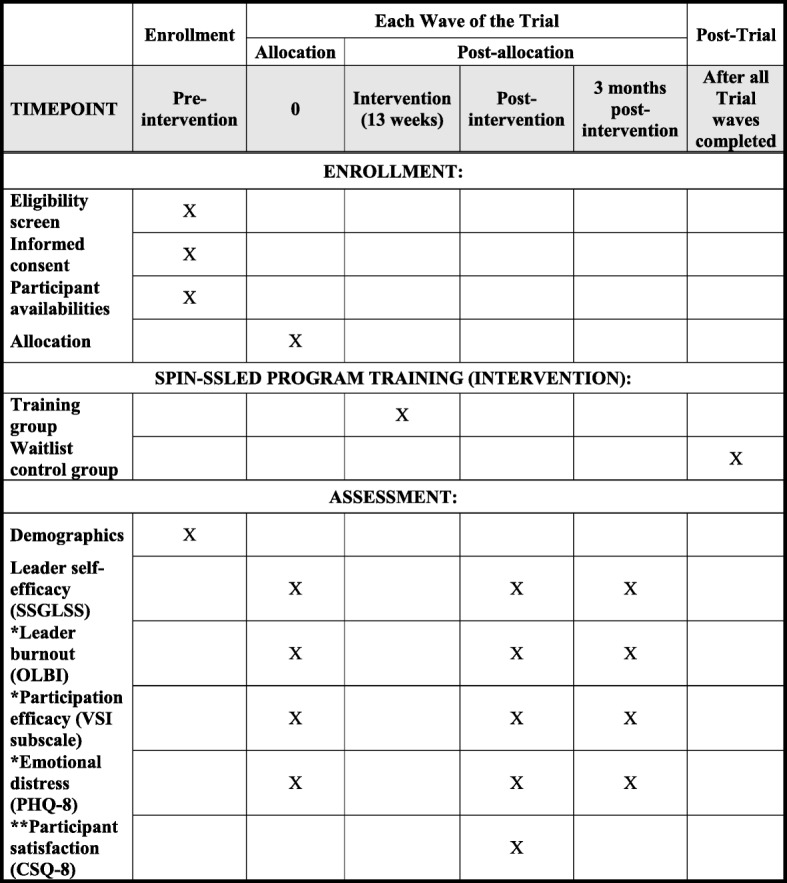
Schedule of enrollment, interventions, and assessments for each of the five study waves of the SPIN-SSLED trial

Interested leaders who provide consent for participation will be entered into different pools based on their availabilities and taking into consideration time zone differences. Figure 1 illustrates the schedule of enrollment, interventions and assessments for each of the five study waves of the SPIN-SSLED trial. For each wave, a third-party centralized randomization service, the Griffith Randomisation Service [86], will randomly select the leaders to be allocated to the intervention and waitlist trial arms. External centralized randomization will ensure that the allocation sequence is concealed and not able to be influenced by study investigators [87]. For each of the three new training groups within each wave, SPIN-SSLED personnel will provide the Griffith Randomisation Service with an anonymized list of participants (only ID numbers will be provided) who could participate in the training group based on their day and time availabilities. For each of the three groups, the service will randomly select 12 participants from the pool of enrolled group leaders available during the designated day and time for the group and will randomly allocate 6 to the training group and 6 to the waitlist group using block randomization. To maximize sharing of experiences in groups, we will limit the number of candidate group leaders without prior experience to 1–2 per training group, depending on the number of candidate leaders who enroll (to be determined). Thus, the maximum number of candidate leaders per 12 selected will be either 2 or 4, and randomization will be stratified by existing and candidate leaders.

All 12 leaders (training group = 6, waitlist = 6) will receive an email invitation including a clickable link to the online survey platform Qualtrics, where they will be asked to complete the study baseline measures. This email will also communicate participants’ assignment to the training program or waitlist control. A second email will be sent to leaders allocated to the training group with the date and time of their first training session, the topic of the first session, the program manual, and information on how to log in to the videoconferencing system and online chatroom. Calls will be made if measures are not completed. Similar procedures will occur post-intervention and 3 months post-intervention.

### Blinding and protecting against sources of bias

A potential concern is that participants will not be blinded to intervention status. In most pragmatic trials of training, education, or behavioral interventions, as in the SPIN-SSLED trial, participants cannot be blinded. This is understood as part of the response to being offered a treatment, similar to what occurs in clinical practice [62]. Since there is no blinding of participants or trainers, there is not a protocol for unblinding.

A second concern relates to the potential for contamination if participants randomized to the SPIN-SSLED Program share learning material with participants in the waitlist control. It is not likely that material would be shared between leaders from different support groups. Nonetheless, to attempt to minimize the influence of possible contamination, we will explain this concern to participants in the training arm of the trial and ask them not to share their material or discuss the training sessions with other group leaders during the trial.

### Data collection and management

Outcome measures will be completed using the online surveying tool Qualtrics. This method was used in the SPIN-SSLED feasibility trial, and data completion was 100% for all variables at baseline and post-trial. Limits on eligible values that can be entered will be set in Qualtrics to reduce erroneous entries. Once the online survey data are collected, the data will be exported to the statistics software program, IBM SPSS. Members of the study team will check and clean the data using SPSS. All information obtained about the participants during this study will be treated confidentially within the limits of the law. To protect the participants’ privacy, upon inclusion in the SPIN-SSLED trial, a unique participant identification number will automatically be assigned to each participant. An encrypted database will be created for the SPIN-SSLED Program, which includes the participant identification number and name. Data security measures in place at Qualtrics are described in the Qualtrics security statement [88]. Information obtained from the survey and video recordings of the training sessions used to evaluate fidelity to the program will be kept for 10 years on encrypted hard drives. Access to the data during the trial will be limited to the study investigators. Once trial results are reported, de-identified data will be made available upon reasonable request. No biological specimens will be collected.

### Data analysis

Analyses will be conducted by a statistician blind to trial arm allocation. For the primary outcome analysis (SSGLSS post-intervention), we will use an intent-to-treat analysis that compares all patients randomly allocated to the SPIN-SSLED Program to all patients allocated to the waitlist control. Intervention effect will be estimated using a linear mixed model, adjusted for baseline SSGLSS scores. The model will include a random effect to account for clustering of participants in the training groups, but not for participants in the waitlist control arm, because there is no clustering in the control arm [61, 62]. We will investigate the effects of missing data using multiple imputation analysis. As a secondary analysis, we will examine SSGLSS scores post-intervention adjusted for baseline SSGLSS scores, age, sex, whether or not the leader has SSc, and candidate versus experienced leader status. The SSGLSS at 3 months post-intervention will be analyzed similarly to the primary and secondary analyses.

Analyses of leader burnout, participation efficacy, and emotional distress outcomes will only include experienced leaders because candidate leaders would not yet have experienced burnout, participation efficacy, or emotional distress due to the burden of leading a group. These outcomes will similarly be analyzed (1) controlling for baseline scores only and (2) controlling for baseline scores, age, sex, and whether or not the leader has SSc. Statistical significance for all analyses will be determined based on two-sided α = 0.05.

### Data monitoring

The trial will be overseen by the SPIN Steering Committee along with the trial investigators and the Support Group Advisory Team. The Steering Committee will provide scientific direction for the RCT and will meet periodically to assess its progress. It will be responsible for RCT protocol execution, routine monitoring of data quality, and will meet semi-annually to discuss recruitment and retention and to assess that the trial is meeting key milestones consistent with the timeline.

### Risks and potential benefits of participating in the SPIN-SSLED trial

Participation in the SPIN-SSLED trial will involve weekly online training sessions and completion of online measures. We do not anticipate any safety concerns with the use of the SPIN-SSLED program, although if there are any adverse events, they will be reported to the local research ethics committee. Although it is hypothesized that the SPIN-SSLED Program will improve leaders’ self-efficacy for performing leader tasks, reduce burnout, and reduce emotional distress, it cannot be guaranteed that leaders will receive any benefits from this study. However, information learned from this research may lead to more effective SSc support group leader training programs, which may benefit those living with SSc in the future or people with other diseases. There will be no financial compensation for leaders who participate in the SPIN-SSLED trial.

## Ethics and dissemination

The SPIN-SSLED trial has been approved by the Research Ethics Committee of Centre intégré universitaire de santé et de services sociaux (CIUSSS) du Centre-Ouest-de-l’Île-de-Montréal (#2020–1780). All participants will provide electronic consent prior to taking part in the study. Any modifications to the protocol which may impact on the conduct of the study, including changes of study objectives, study design, patient population, sample sizes, study procedures, or significant administrative aspects will undergo a formal amendment to the protocol. This amendment will be submitted to the Research Ethics Committee for approval and documented in the trial registration.

Our trial team has worked closely with patient organization partners from around the world and with a Support Group Advisory Team to design each stage of preliminary research, the SPIN-SSLED Program, and the SPIN-SSLED feasibility and full-scale trials. To the best of our knowledge, once tested, SPIN-SSLED will be the only peer support group leader training program that has been evaluated in a well-conducted RCT in any disease. Our SSc organization partners plan to implement the program post-trial to train and certify peer support group leaders, and the trial team will work with them to do this. Beyond SSc, the SPIN-SSLED Program will be easily adapted for use in other diseases.

## Trial status

This is the first version of the protocol, finalized on 15 July 2019. Recruitment will begin 1–2 months prior to the beginning of the trial (July and August 2019), which is currently planned for September 2019.

## Supplementary information

**Additional file 1.** SPIN-SSLED Program module overview.

## Data Availability

All data and materials will be provided upon reasonable request. SPIN-SSLED Program materials are copyrighted under a Creative Commons Attribution-NonCommercial-ShareAlike license.

## Abbreviations

CONSORT: Consolidated Standard of Reporting Trials
CSQ-8: Client Satisfaction Questionnaire-8
IBM SPSS: International Business Machines Corporation Statistical Package for the Social Sciences
ICC: Intra-class correlation coefficient
OLBI: Oldenburg Burnout Index
PHQ-8: Patient Health Questionnaire-8
PN-RCT: Partially nested randomized controlled trial
RCT: Randomized controlled trial
SD: Standard deviation
SPIN: Scleroderma Patient-centered Intervention Network
SPIRIT: Standard Protocol Items: Recommendations for Interventional Trials
SSc: Systemic sclerosis
SSGLSS: Scleroderma Support Group Leader Self-efficacy Scale
SSLED: Scleroderma Support group Leader EDucation Program
UK: United Kingdom
VSI: Volunteer Satisfaction Index
