# Supplementary material for: Protocol for a partially nested randomized controlled trial to evaluate the effectiveness of the Scleroderma Patient-centered Intervention Network Support Group Leader EDucation (SPIN-SSLED) Program
Source: Trials. 2019 Dec 12;20:717. doi: 10.1186/s13063-019-3747-z (PMC6909446; doi:10.1186/s13063-019-3747-z)
Supplement: Supplementary file 1 — Additional file 1. SPIN-SSLED Program module overview. [file 13063_2019_3747_MOESM1_ESM.docx]

**SPIN-SSLED Program Module Overview**

| Module Title | Module Description |
| --- | --- |
| 1. The Support Group Leader’s Role | This module discusses the benefits of being a support group leader, the expectations of what the role of leader involves (e.g. facilitation of meetings and interactions but not giving medical advice), and tips for being an effective and supportive leader. |
| 1. Starting a Support Group | This module discusses the purpose of a support group, what people with scleroderma hope to gain from support group, why some don’t attend, establishing leadership (e.g. one leader, co-leader), membership (e.g. patients only, open to family, and friends), logistics of starting a group (e.g. time, place and meeting duration). |
| 1. Structuring Support Group Meetings | This module discusses formatting group meeting and how to successfully integrate both educational activities with emotional and practical support for members, setting up a meeting agenda. |
| 1. Scleroderma 101 | This module shows a filmed conference by a physician specialized in scleroderma who explain the different types of scleroderma, symptoms, causes, treatments, and alternative approaches. The module also includes tips to evaluate credibility of information sources on the Internet. |
| 1. Successful Support Group Culture | This module discusses the importance of establishing expectations and guidelines for the support group with members, the importance of confidentiality, how to create and maintain positive and productive support group culture using (1) encouraging statements, (2) open-ended questions, (3) body language, (4) linking similar experiences between members, and (5) summarizing discussions. This module uses video vignettes to illustrate these techniques. |
| 1. Managing Group Dynamics | This module discusses managing difficult support group dynamics such as members who are “quick fixers”, overly talkative, how to maintain a positive group environment, conflict management and resolution for minor and larger issues. The topics discussed also include overly shy members, chronically negative members and members that bring unsubstantiated, potentially misleading medical information to the group. This module uses video vignettes to illustrate these techniques. |
| 1. Loss and Grief: The Support Group Leader | This module defines loss, bereavement, grief and mourning. The module discusses the styles of processing loss and grief, healing strategies and how to deal with loss and grief as a support group leader. The importance of creating a loss and grief plan for the support group is also discussed. |
| 1. Loss and Grief Scleroderma: Supporting Group Members | The module discusses how grief may be experienced in newly diagnosed members, common cognitive and emotional reactions that people can experience in response to a diagnosis as well as reaching acceptance and adaptation with respect to your diagnosis. |
| 1. Advertising and Recruiting for the Support Group | This module discusses to advertise and promote a support group, how to recruit new members for support groups on ongoing basis, advertising through patient organizations and strategies to retain members. |
| 1. The Continuity of the Group | This module discusses the importance of understanding and overcoming reluctance in seeking feedback, the importance of feedback in the support group experience, how to obtain and respond to feedback, how to identify reasons why members may stop attending meetings and strategies to help maintain membership, how to keep members engaged and move your support group forward by making changes. |
| 1. Supporting Yourself as a Leader | This module discuses understanding what leader burnout is, understanding why it can happen and what the warning signs are, understanding the best way to address burnout including identifying methods of coping, understanding at what point it may be best for a leader to step down from his or her role, strategies to prevent experiencing leader burnout. |
| 1. Remote Support Groups | This module discusses the benefits of an online support group, finding the right technology, scheduling and programming, advertising and reaching your target audience, tips for successful online meetings. |
| 1. Transitions in Support Groups | This module discusses how to handle transitions in support group leadership, discussing leader’s experiences with the SPIN-SSLED Program and some meaningful takeaways. This module also discusses the transition from the weekly program training sessions to the optional monthly teleconference meetings. |
